# Nominating Committee

**Published:** 1867-07

**Authors:** 


A recess of 10 minutes was taken, to enable the delegates from each county represented to select one of their own number, to constitute a committee, for nominating officers and standing committees for the ensuing year. On being called to order, the following members had been selected to constitute the Nominating Committee:—

Drs. W. S. Edgar, of Morgan Co.
Louis Watson, of Adams Co.
D. W. Young, of Kane Co.
D. S. Jenks, of Kendall Co.
J. C. Corbus, of LaSalle Co.
C. Goodbrake, of DeWitt Co.
J. F. Potts, of Peoria Co.
J. T. Frazer, of Fayette Co.
R. N. Isham, of Cook Co.
J. S. Whitmire, of Woodford Co.
M. Reese, of Knox Co.
H. B. Buck, of Sangamon Co.
S. T. Trowbridge, of Macon Co.
T. F. Worrell, of McLean Co.
G. W. Albin, of Cumberland Co.
O. Q. Herrick, of Edgar Co.
D. L. Jewett, of Iroquois Co.
S. B. McGlumphy, of Logan Co.
T. D. Washburn, of Montgomery Co.
J. L. White, of Jersey Co.
H. W. Boyd, of Madison Co.
F. H. VanEaton, of Greene Co.
B. K. Shurtleff, of Lee Co.

The Secretary read a letter from Dr. Taggert, of Cairo, explaining his inability to attend the present meeting. Also, a letter from Dr. R. G. Bogue, of Chicago, member of the

Special Committee on Deformities of the Spine and Joints, explaining the non-appearance of a report by the Committee, and asking further time, with the addition of Dr. F. O. Earle, of Chicago, to the Committee.

On motion, the request of Dr. Bogue was granted, and the Committee continued another year.

Dr. H. A. Johnson moved that the amendment to the Constitution, proposed last year, *granting to permanent members the right to vote, the same as delegates*, be taken from the table and adopted. The motion was sustained by a vote of 24 yeas and 1 nay; the members of the Nominating Committee being absent from the room.

Dr. DeLaskie Miller moved the adoption of a pending amendment to the Constitution, fixing the regular annual meetings of this Society on the *Third Tuesday in May*, in each year. The amendment was adopted by a vote of 32 yeas, 0 nays.

Dr. J. Adams Allen, Chairman of the Standing Committee on Practical Medicine and Epidemic Diseases, stated that the report consisted chiefly of two papers; one on Cholera, as it prevailed in Chicago in the summer of 1866, written by Dr. Marsh, of Chicago; another on Diseases in the Interior of the State, by Dr. L. T. Hewins, of Loda.

The first of these papers was made the special order for consideration at 2 o'clock P.M.

